# A Finite Element Analysis for Improvement of Shaping Process of Complex-Shaped Large-Size Silicon Carbide Mirrors

**DOI:** 10.3390/ma14154136

**Published:** 2021-07-25

**Authors:** Ling Qiao, Jingchuan Zhu, Yingnan Wan, Congcong Cui, Ge Zhang

**Affiliations:** 1 School of Material Science and Engineering, Harbin Institute of Technology, Harbin 150001, China; qiaoling_1993@163.com (L.Q.); 15134572733@163.com (Y.W.); 2 Key Laboratory of Optical System Advanced Manufacturing Technology, Chinese Academy of Sciences, Changchun 130033, China; zhangge-ciomp@126.com; 3 Changchun Institute of Optics, Fine Mechanics and Physics, Chinese Academy of Sciences, Changchun 130033, China

**Keywords:** finite element analysis, gel-casting process, silicon carbide, defects, loading speed, exhaust hole

## Abstract

In the gel-casting process, the proper selection of technological parameters is crucial for the final quality of a green body. In this work, the finite element method is used to investigate the mold characteristics in the gel-casting process, and the typical flow behaviors under different conditions are presented. Based on the distribution characteristics of temperature, pressure and flow field of gel polymer, the simulated results provide some possible reasons for the generation mechanisms of defects. Then, a series of simulations were performed to investigate the effect of process parameters on the molding quality of green gel-cast bodies. The results show that the decreasing loading speed can effectively reduce the number of defects and improve the molding quality. In addition, this paper presents a new technique by applying the exhaust hole to decrease the number of defects and, hence, improve structural integrity. The influence of the loading speed on the mold characteristics is well understood for the gating system with an exhaust hole, which suggests to us appropriate parameters for optimizing the molding design. This work provides a theoretical basis to explicate the generating mechanism of defects involved in the gel-casting process and acquires an optimized technique to produce a silicon carbide green body.

## 1. Introduction

Gel casting is a promising technology for manufacturing advanced structural ceramic bodies that have been invented in recent years [1,2]. The gel-casting process usually involves the gelation and densification of slurry. Compared with the traditional ceramic forming processes, e.g., slip casting, tape casting and dry pressing, the potential advantages of gel casting include faster casting, favorable uniformity and high green body strength, which allows for fabricating of complex shapes and machining in green state [3,4]. As well as these preferred characteristics, gel casting avoids slumping during binder removal and enables the final green body with thin wall structures and high solid loading [5], which has successfully generated wide application in various materials, such as alumina, silicon nitride, silicon carbide. In recent years, the importance of the forming process has been gradually realized and the technical parameter optimization of gel systems has become an important subject in scientific research [6,7,8].

As the most promising material for space-based lightweight optical mirror, silicon carbide has become a favorite topic for analysis due to its outstanding properties, such as excellent specific stiffness, low density and thermal expansion coefficient, high elastic modulus and thermal stability, etc. [9]. Despite empirical research focused on the preparation process and the corresponding optical properties, detailed information on the forming process has not been paid enough attention. The ceramic optical mirrors are usually difficult to fabricate with complex geometrical shapes and a high precision requirement, which limits their engineering applications [10,11,12]. Given this, it is deemed necessary to find an appropriate technique to fabricate a lightweight SiC ceramic optical mirror. For example, Yui et al. [13] have attempted to optimize the manufacturing process of the lightweight large silicon carbide mirror, from designing the structure and mixing the raw material to final polishing. Zhang et al. [14] utilized the finite element analysis method to investigate the stress and displacement distribution of the large-scale fabrication of the lightweight Si/SiC ceramic composite optical mirror under a factual condition. As is known, gel casting is a near-net-shape forming method to prepare complex ceramics part at a low cost. The homogenous distribution of the polymer using the gel-casting technique contributes to the higher strength of the SiC materials. So far, many examples involved in the application of the gel casting in forming silicon carbide green samples can be found in the reported literature [15,16,17,18,19,20,21,22,23]. Tian et al. [24] successfully prepared the SiC ceramics using the gel-casting process and proposed a pretreatment and surface modification method to avoid the problem that Isobam is difficult to crosslink and gelling with non-oxide ceramics. Jin et al. [25] developed a novel ethylene glycol-based gel cast to prepare highly porous SiC ceramics, and the strength of green bodies was effectively increased. However, it is worth noting that the gel-casting technologies cannot completely eliminate the defects caused by the thermal non-uniformity. The gas trapping and shrinkage porosity tend to generate the cracks and defects on the surface and inside gel-cast green bodies, thus then resulting in a failed casting. Therefore, cost-effective methods and optimized process parameters are in desperate need for reducing defects to the ceramic body.

Knowing that the gel-casting technique critically relies on the loading speed, temperature, cooling method and design of gating system, accordingly the proper selection of the process parameter is crucial for the quality of a green body. Moreover, studies on the evolution and control of defects in gel-casting ceramic bodies are also of great importance in improving the forming quality. Nowadays, the finite element method (FEM) has been reported as a common tool for process optimization in terms of time and cost savings [26,27]. With the aid of FEM, the temperature field and flow field in different conditions can be simulated and analyzed briefly [28]. In this paper, such efforts have been made for design and optimization of the gel-casting process. SiC was used as the material of the optical mirror, and the FEM technique was applied to construct the thermal–mechanical coupling model according to the requirement of the system’s technology. Measurements of the viscosity were performed on the slurry for qualitative FEM analysis. The distribution characteristics of the temperature, the pressure, and fluid velocity throughout the molding process were studied using this model. The objective of this paper is to develop a gel-casting process for fabricating an SiC ceramic optical mirror and to examine the role of processing parameters with regard to the molding quality. The generating mechanism and eliminating solutions of defects appearing on green bodies are also important aspect to discuss in this work. Finally, the influence of the exhaust hole was studied for the purpose of optimizing the techniques for the casting system. This work allows us to obtain the reasonable technique parameters and provides some fundamental guidance for the fabrication of a lightweight SiC ceramic optical mirror.

## 2. Computational Method

### 2.1. Materials and Modeling

**Figure 1:**
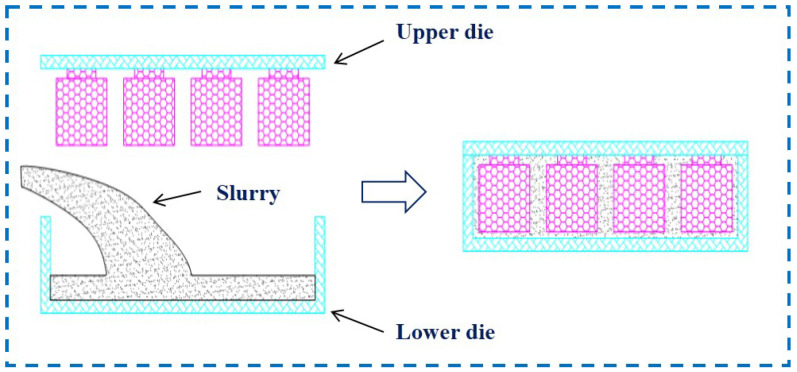
Flowchart of the forming process.

Figure 1 presents the schematic illustration of the gel-cast process, which can be used to explain our model. Generally, gel casting of SiC combines complex chemical and physical processes, which can be described briefly [29]: the preparation of slurry involves the submicrometer-sized SiC ceramic particles together with a certain proportion of gel initiators, catalysts, etc. Herein, the average particle size of SiC ceramic particles is 12.8 μm, and the solids content is about 48 Vol.%. Prior to gel casting, the slurry is sufficiently stirred, ultrasonicated and degassed under vacuum. Gel casting is carried out by pouring the mixing slurry into a complex-shaped cavity and continues with in situ polymerization of ceramic particles. After removal from the mold, the structural ceramic green with precise-shaped characteristics and high mechanical strength can be obtained by drying, and finally machined. In view of that, the mold is divided into two parts, upper and lower die, which are initially separated. After the SiC slurry pouring into the lower die, the upper die is carried out to close the mold and then the slurry fills the cavity and solidifies to realize the formation of the mirror.

**Figure 2:**
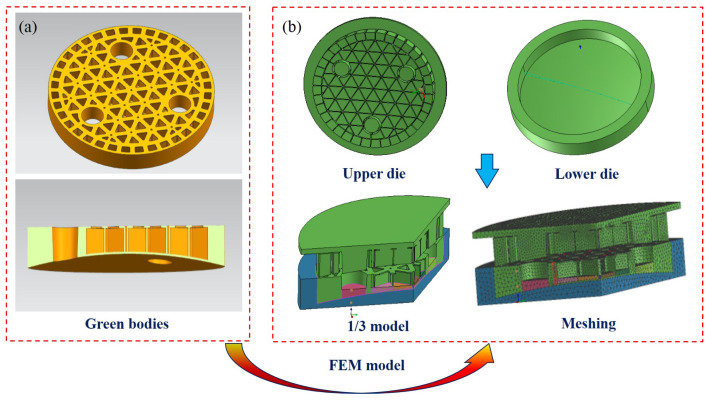
(**a**) 3D models of green bodies; (**b**) FEM model.

The complex-shaped optical mirror is presented in Figure 2a, which exhibits a circular shape with a diameter of 420 mm with three central holes of 104 mm in diameter. Following this description, the thermal–mechanical coupling model is constructed using ProCAST software. Due to the symmetry of the green body, the model can be simplified into 1/3 of the real model to reduce the computation burden and improve the operation speed. Many studies have confirmed that the quality of the finite element mesh is one of the important factors determining the precision and accuracy of results. Thus, the visual mesh is utilized to mesh the model, and then the grid data are imported to ProCAST for the subsequent simulation. Particularly in this case, the mesh generation starts with face mesh and then follows by the adaptive volume mesh. The finite element mesh includes not only basic geometry, such as rectangle and triangle shapes, but also closed high-order curves and even various combined shapes. The appropriate size should put a special emphasis in different parts, which is closely associated with the calculation accuracy [30,31]. The finer mesh is generated at the contact part between upper die, and the fairly coarse mesh is determined on the uncontact parts. Finally, the solution domain is divided by adopting 3D mesh generator with 439,945 domain elements and 74,449 2D mesh elements. The simplified geometric structure of the green body used in the finite element simulation are shown in Figure 2b accompanied by the details of mesh dividing.

### 2.2. Boundary Condition and Physical Parameters

In addition to the introduction of numerical simulation methods and the construction of green body models, the setting of the boundary conditions and physical parameters are specially described in this part. Within the framework of ProCAST, the flow analysis module plays a critical role in unveiling the flow mechanism in porous media. The coupling flow field of the free surface in the unsteady filling process can be calculated via the Navier–Stokes equations and the gas trapping of slurry can be particularly explored in depth. The flow field model proposed in this work complies with the basic conservation equation, such as mass conservation equation, Equation (1), momentum conservation equation, Equation (2), and energy conservation equation, Equation (3), which have been given as follows.

(1) $$\begin{array}{c} \frac{\partial u_{x}}{\partial x}+\frac{\partial u_{y}}{\partial y}+\frac{\partial u_{z}}{\partial z}=0 \end{array}$$

(2) $$\begin{array}{c} \frac{\partial u}{\partial t}=g_{x}\frac{1}{\rho }+\frac{\partial p}{\partial x}\left(u\frac{\partial u}{\partial x}+v\frac{\partial u}{\partial y}+w\frac{\partial u}{\partial z}\right)+v\left(\frac{\partial^{2}u}{\partial x^{2}}+\frac{\partial^{2}u}{\partial y^{2}}+\frac{\partial^{2}u}{\partial z^{2}}\right) \end{array}$$

(3) $$\begin{array}{cc} \frac{\partial (\rho T)}{\partial t} & +\frac{\partial (\rho T_{u})}{\partial x}+\frac{\partial (\rho T_{v})}{\partial y}+\frac{\partial (\rho T_{w})}{\partial z}\\ \; & =\mu \left(\frac{\partial^{2}w}{\partial x^{2}}+\frac{\partial^{2}w}{\partial y^{2}}+\frac{\partial^{2}w}{\partial z^{2}}\right)-\frac{\partial p}{\partial z}+\rho g_{z} \end{array}$$

where, *u*, *v* and *w* represent the velocity components in *x*, *y* and *z* directions, respectively. *P* represents the pressure. gx, gy, gz represent the component of gravity acceleration. μ represents the viscosity of fluid and *t* represents the temperature. λ represents the thermal conductivity of fluid, and *F* represents volume fraction of fluid. Specifically for an incompressible fluid, the continuity equation takes the following form.

(4) $$\begin{array}{c} D=\frac{\partial u}{\partial x}+\frac{\partial v}{\partial x}+\frac{\partial w}{\partial x} \end{array}$$

The volume of the fluid function equation can be expressed as

(5) $$\begin{array}{c} \frac{\partial F}{\partial t}+u\frac{\partial F}{\partial x}+v\frac{\partial F}{\partial y}+w\frac{\partial F}{\partial z}=0 \end{array}$$

In this model, the green body is assumed to be a porous medium, and the Carreau–Yasuda model is used to describe the viscosity of slurry, Equation (6).

(6) $$\begin{array}{c} \eta =\eta_{\infty }+\left(\eta_{0}-\eta_{\infty }\right){\left[1+{\left(\lambda \cdot \gamma \right)}^{a}\right]}^{\frac{n-1}{a}} \end{array}$$

where $\eta_{\infty }$ presents the ultimate strain rate viscosity, $\eta_{0}$ presents strain free viscosity, λ presents the phase inversion, *a* is the exponential coefficient, and *n* is the Yasuda coefficient.

On the other hand, the solution of the flow field needs to deal with temperature, velocity and pressure such that the accuracy of the simulated results is strongly depended on the setting of the boundary condition. At the initial stage of the filling process, the initial boundary conditions can be described as follows:

(7) $$\begin{array}{c} \Theta \left(\theta ,0\right)=\Theta_{0}\left(\theta \right) \end{array}$$

where Θ denotes the dependent variables and θ denotes the independent variable, such as temperature, velocity and pressure. Then, the boundary condition at the end of the filling process can be expressed as a time-dependent functions.

(8) $$\begin{array}{c} \Theta \left(\theta \right)=\Theta_{\Gamma }\left(\theta \right)f\left(t\right) \end{array}$$

where Γ is a specific boundary, and f(t) is a time function. As a result, the non-Newtonian flow models can be constructed based on the abovementioned governing equations, which are coupled and solved through FEM.

**Figure 3:**
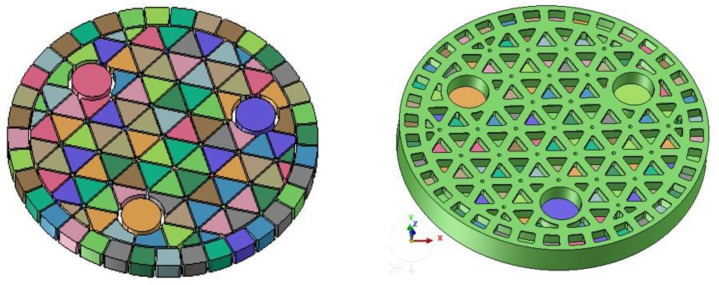
The filler and initial slurry model.

**Figure 4:**
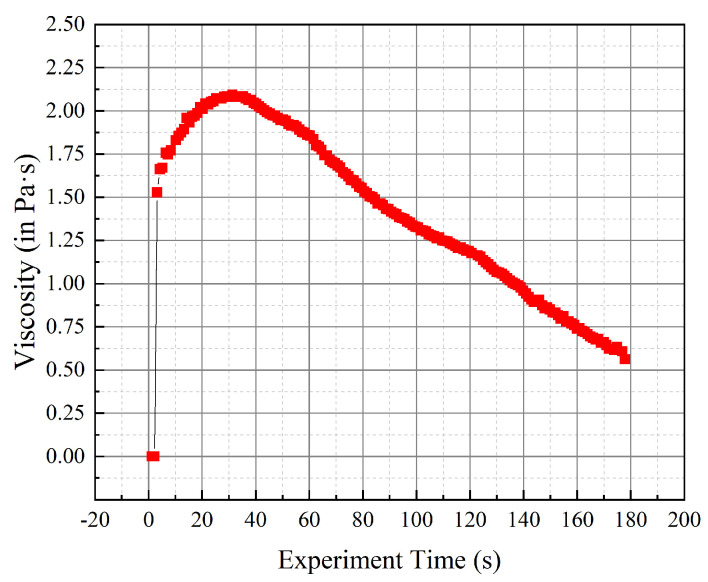
The viscosity of SiC by experiments.

Given this, the gel-casting process of SiC can be effectively analyzed by FEM depending on the materials module. Especially note that the initial model is highly important for the accuracy of results, such that its shape and height should be paid particular attention. Assuming that the initial filling rate is 40%, the filler and initial slurry model are specifically illustrated in Figure 3. In terms of the physical properties, the parameters, such as the melting point, density and solidus temperature, etc., are regarded as constants in the gel-casting process. Meanwhile, the viscosity varies with time, which should be primarily considered in the gel-casting process. Herein, the SiC powder (purity > 99%) was used to prepare the slurry, and the viscosity of slurry was measured via the viscometer. Figure 4 plots the time-dependent viscosity of SiC by means of experimental measurements. As known, the slurry is the typical non-Newtonian fluid. The shear rate varies with time, as well as the shear stress. At the beginning, the shear stress and viscosity of the slurry increase with the increase in shear rate. With the experimental time, when the shear rate exceeds a certain value, the viscosity of the slurry keeps at a certain value and then decreases slowly, showing the characteristics of shear thinning and pseudoplastic fluid. In addition, the existence of hydrophilic polymer in the dispersion system will destroy the network and branch chain structure of the polymer owing to the shear stress, ultimately resulting in the decrease in viscosity. Eventually, the measured viscosity can be applied to explore the effect of the viscosity on the gel-casting process.

The gravity boundary conditions are applied to the green body in the direction of the negative z-axis. Given the contact and interface between each part of the model, the interface type is determined between the upper die, the green body and lower die. At the initial stage, the Ncoincinterface is determined in terms of non-contact condition between the upper die and green body. The Coincinterface is selected since it is always in complete contact for the green body and lower die. Besides this, some key physical parameters are considered in this case primarily, including the maximum number of steps, the total simulation time, the maximum time step of filling process, the free surface model and gas model, etc., which are summarized in Table 1. In general, for the physical properties of materials, the viscosity is mainly considered, while other thermophysical parameters such as solid temperature, melting point and boiling point are not the main factors for the injection molding process. Considering that the database of ProCAST software does not contain the thermo-physical properties of the SiC gels, a similar material with the melting point of 445–630 °C, which is involved in the database of ProCAST software, is selected and the simulation is performed at 435 °C. Based on the given boundary condition, detailed analysis is carried out on the dynamic filling process. Particularly, the presented speed control in the filling process includes two steps: (1) determining the loading speed of 10 mm/s for upper die when it is yet to be in contact with the slurry, and (2) decreasing to 5 mm/s once the upper die touches the slurry. Given this physical model, the generating mechanism of defects appearing on green body can be analyzed by extracting the pressure and fluid field distribution.

## 3. Results and Discussion

### 3.1. Analysis of Defects in Gel-Casting Process

**Figure 5:**
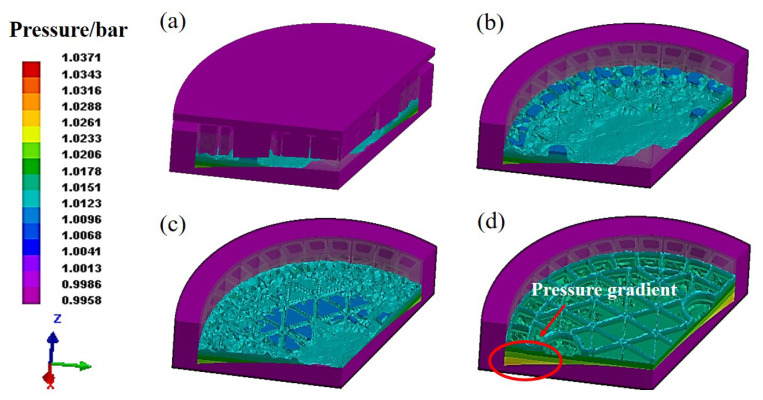
The pressure distribution during gel-casting process at (**a**,**b**)19.07 s, (**c**) 31.81 s, (**d**) 34.65 s.

Figure 5 plots the pressure distribution during the gel-casting process, as well as the characteristics of gel polymer. The liquid is not in the state of horizontal extrusion but will fluctuate locally with extrusion filling. Figure 5a presents that the upper die comes into contact with the slurry. As Figure 5b,c illustrate, the difference in the slurry concentration can be observed between the center and the edge of the green body. In view of the pressure contour, there exists the non-homogeneous pressure distribution where the deep blue colors appearing on the surface seem to exhibit lower pressure. Specifically in the direction of gravity, the pressure contours from Figure 5d imply a gradient distribution from the bottom of the slurry to the top, which varies from the maximum to the minimum. It can be observed that the distribution of the slurry becomes more homogeneous as the filling process continues. After the ceramic particles and resin network fulfil the green body, it seems that the green body exhibits nearly uniform distribution on the surface.

**Figure 6:**
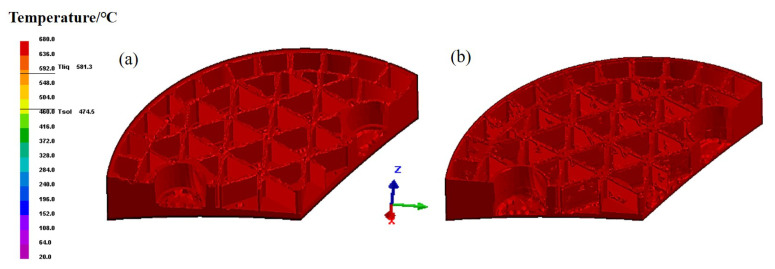
Temperature distribution in the filling process. (**a**) 38.56 s, (**b**) 38.91 s.

**Figure 7:**
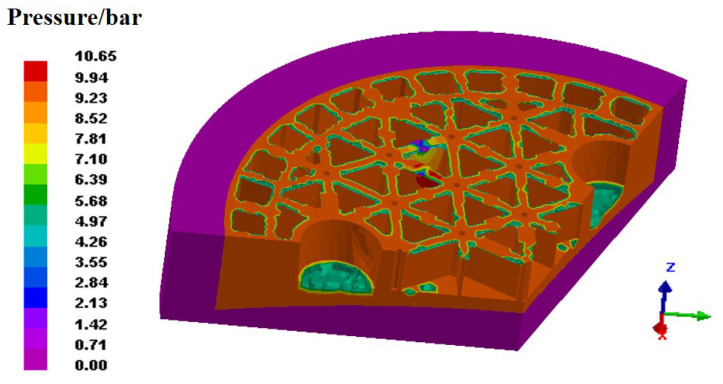
The pressure distribution after the filling process.

Reducing the number of defects in the filling process is significant for the final quality of formed components. Hence, our focus is to understand the formation mechanism of various defects that seriously affects the following design and production. The porous media heat transfer model is applied to identify the temperature distribution of the green body, as the temperature contours shown in Figure 6. By comparing the surface morphology in Figure 6a,b, many obvious defects, such as cracks and pores, can be observed on the surface with the filling process. In view of the temperature contours, the generally uniform temperature distribution can be identified on the surface of body, as well as the cross sections. Accordingly, the formation of defects is rarely dependent on the temperature gradients in the body. Given the complex geometrical model, nevertheless, the capillary effect exists consequentially owing to the irregular-shaped upper die. Since the flow behavior of the slurry continues, gas appears even though the upper die stops pressing down. Then, a large amount of gas trapping probably occurs and remains on the surface of the green body. To discuss this in depth, Figure 7 plots the pressure distribution in the filling process. The lower pressure can be observed on the surface of green body as well as the edge of vertical ribs, which is probably related to the gas trapping. The gas in the slurry moves constantly with the varying pressure, inevitably resulting in the various defects on the forming structure after the filling process.

**Figure 8:**
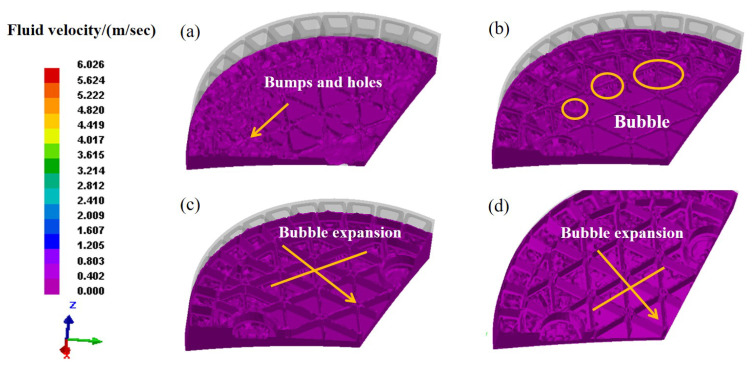
Fluid velocity contours in filling process. (**a**) 33.12 s, (**b**) 34.24 s, (**c**) 35.01 s, (**d**) 36.92 s.

For a better understanding, Figure 8 shows the fluid velocity contours in the filling process. The results imply a uniformity of fluid velocity distribution in the entire filling process. At the initial stage, Figure 8a indicates the uneven and rough surface which is full of bumps and holes. As the upper die pressed down, a great number of pores and bubbles could be observed in Figure 8b. It is speculated that the obstructed fluidity of slurry and the uneven filling process are the main factors for producing so many bubbles. Under pressure, the bubbles tend to develop and simultaneously move upwards to form defects on the ribs. Figure 8c presents the growing bubbles and the eventually resulted defects after the filling process. In general, the proposed FEM model can effectively describe the pyrolysis behavior observed in the entire filling process, including the depressurization process of the upper die, the fluctuation of slurry after contacting with the upper die, etc. The gas trapping involved in the slurry is considered as one of the major reasons for the generation of various defects.

### 3.2. Influence of Process Parameters on Defects in Gel-Casting Process

For the gel-casting process, many parameters, such as the physical property parameters of mold, temperatures, loading speed, pressure, etc., play a critical role in the molding characteristics [32,33]. The selection of reasonable loading speed is particularly important. In the current research, adjusting loading speed and optimizing the design of casting system might be utilized to avoid defects and improve the resultant green bodies. A detailed investigation is presented to identify their exact influences to gain deep insights into the gel-casting process. The great importance of the optimized gel-casting characteristics will be revealed in this section.

**Figure 9:**
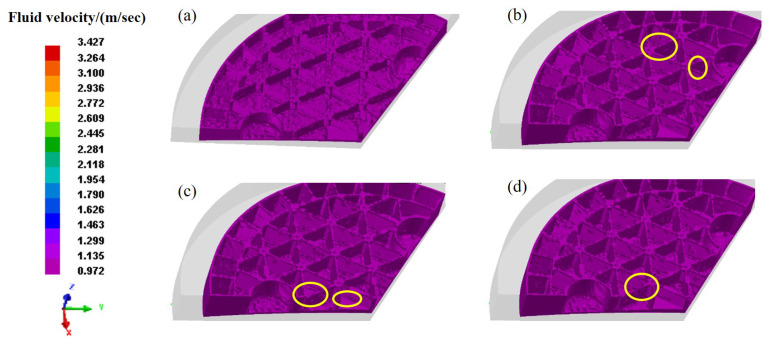
Fluid velocity of green body at different pressing speeds. (**a**) 20 mm/s; (**b**) 10 mm/s; (**c**) 5 mm/s; (**d**) 1 mm/s.

To capture the influence of loading speed on molding, a scheme of different loading speeds (1 mm/s, 5 mm/s, 10 mm/s and 20 mm/s) can be designed to find a reasonable technical parameter. Herein, two aspects are theoretically included: the flow behavior and pressure distribution of slurry in the gel-casting process. Figure 9 shows the fluid velocity distribution at different loading speeds. Evidently, under the determined loading speeds, there exists a large quantity of bubbles and defects in terms of the molding characteristics. At the loading speed of 20 mm/s, Figure 9a shows a rough model with many structural defects, not only on the surface but also on the ribs. Specifically as the loading speed decreases, the number of deep defects is remarkably reduced on the ribs by comparing the molding characteristics of Figure 8a,b. As the loading speed decreases to 5 mm/s and 1 mm/s, it can be found from Figure 9c,d that little variation can be observed on the shapes of the mold contours. Such that the molding quality can not be further improved with the successive decrease in loading speed. As a result, the properly decreasing loading speed can achieve our goal of improving molding quality and avoiding the structural defects.

**Figure 10:**
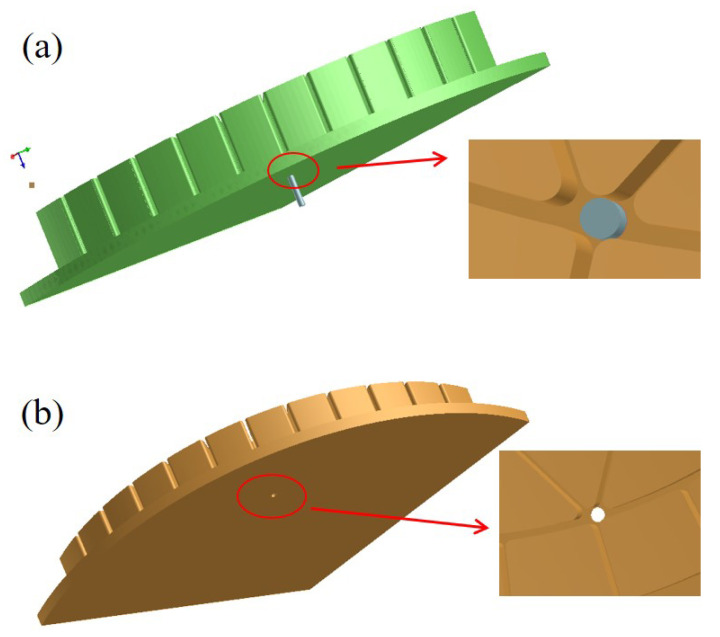
Schematic diagram of exhaust hole location. (**a**) without vent hole, (**b**) with a vent hole.

To further improve the molding quality, the newly developed design on the casting system should be introduced. From the analytical results, the structural defects are mostly caused by gas trapping evolved in the gel-casting process, which seriously damages the final mold. Recognizing this, we attempt to introduce an exhaust hole at the appropriate position on the upper die to reduce the internal gas and bubbles, as illustrated in Figure 9. Specifically in this case, the local fine meshes were particularly used around the exhaust hole to improve the calculating precision. The simulated results by comparing Figure 10a,b are utilized for verifying the superiority of the exhaust hole in molding quality. In order to eliminate the influence of the loading speed, it was set to 10 mm/s in both cases to capture the influence of exhaust holes on the molding features. For gating systems without vent holes, Figure 10a, a large amount of structural defects and bubbles can be found on the surface and even the vertical ribs. However, an apparent variation can be observed on the molding features in Figure 10b, where the model prefabricated a vent hole. It can be deduced that far fewer trap defects are produced in casting by the newly developed system. Owing to the exhaust holes, the molding quality has been significantly improved as a result of the decreasing number of gas trapping. These results can contribute to the further study of process optimization design.

**Figure 11:**
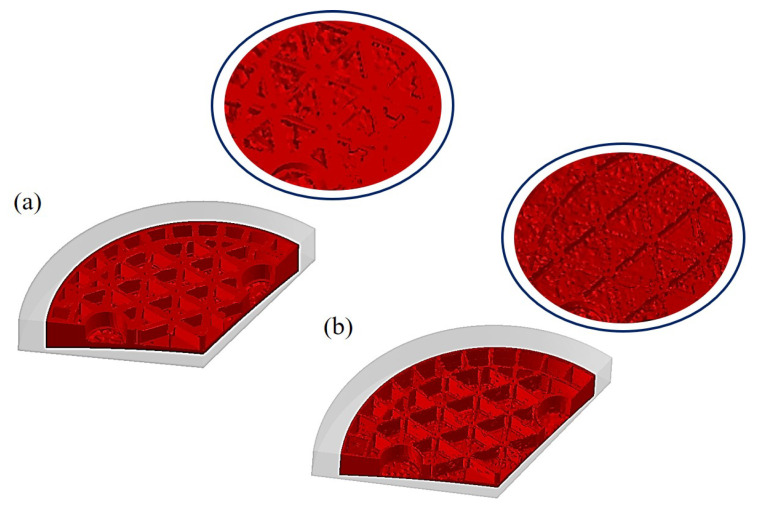
Temperature field for the gating system. (**a**) Without exhaust hole and (**b**) with exhaust hole.

**Figure 12:**
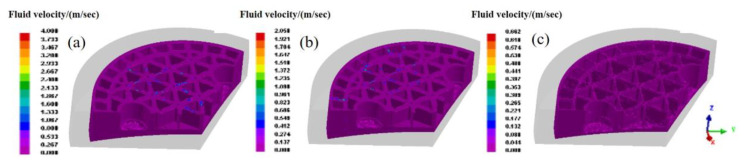
Fluid velocity for gating system with exhaust hole after gel-casting process. (**a**) 20 mm/s; (**b**) 10 mm/s; (**c**) 5 mm/s.

**Figure 13:**
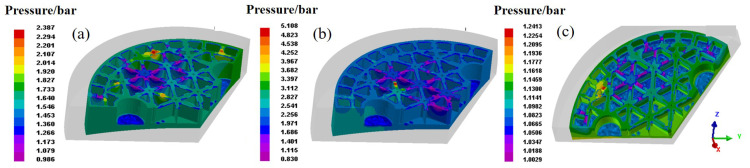
Pressure distribution for gating system with exhaust hole after gel-casting process. (**a**) 20 mm/s; (**b**) 10 mm/s; (**c**) 5 mm/s.

To capture the influence of loading speed for the gating system with the exhaust hole, the forming conditions with loading speed of 5 mm/s and 20 mm/s were further investigated. As the fluid velocity distribution illustrated in Figure 11, it can be inferred that the mold with loading speed of 20 mm/s exhibits improved surface quality rather than that of lower loading speed. Particularly attributed to the exhaust hole, the defects can be reduced effectively. Figure 12 and Figure 13 present the distribution of pressure and fluid velocity with several loading speeds. Evidently, the low pressure region in Figure 12a,b corresponds to the blue part illustrated in Figure 11a,b, indicating the continuing flow behavior of gel polymer even after the filling process. Otherwise, at a low loading speed, the pressure exhibits a significant declining even though associated with the remarkable inhomogeneity. Thus, it can be inferred that an appropriate increase in loading speed can be beneficial for the gating system with the exhaust hole. This combination of process parameters can reduce the structural defects, such as gas trapping, and improve the molding quality.

## 4. Conclusions

In this study, the gel-casting process of SiC has been simulated by ProCAST, including the distributions of pressure, temperature and flow field. The effects of technological parameters on the molding quality and the physical mechanisms of defects are particularly investigated. The following conclusions can be drawn:

(1) The simulated analysis gives a possible reason for the generation mechanisms of defects in the gel-casting process. In general, the severe defects on the forming structure are strongly dependent on the gas trapping involved in the slurry. The varied local pressure contributes to the flow of gas trapping and, hence, results in the severe mold characteristics.
(2) The influence of loading speed on the molding quality was studied. The decreasing loading speed can achieve our goal of avoiding the structural defects and improving molding quality. However, the molding quality would not be further improved when the loading speed decreases below 10mm/s, which may suggest to us appropriate parameters for optimizing the mold design.
(3) The superiority of the exhaust hole provided that the number of defects could be effectively reduced in the final model. Specifically for the newly developed gating system with an exhaust hole, an appropriate increase in loading speed can contribute to an improved molding quality. Thus, this combination of process parameter can reduce the defects, such as gas trapping, and is beneficial for the final molding.

## Figures and Tables

**Table 1:** Simulation parameter setting.

Name	Script	Value	Unit
Nstep	Number of Step	500,000	-
Tstop	Final Temperature	435	°C
DT	Initial Time	0.001	s
DTMAXFILL	Maximum Time Step for Filling	0.01	s
DTMAX	Maximum Time Step	0.5	s
PREF	Reference Pressure	$1.0132\times 10^{5}$	${N/m}^{2}$
LVSURF	Maximum Fill Fraction	1	-
COURANT	Filling Parameter	100	-
WALLF	Wall Slip Parameter	0.99	-

## Data Availability

Confidential.
